# Comprehensive analysis of PPP4C’s impact on prognosis, immune microenvironment, and immunotherapy response in lung adenocarcinoma using single-cell sequencing and multi-omics

**DOI:** 10.3389/fimmu.2024.1416632

**Published:** 2024-07-04

**Authors:** Kaiyu Wang, Bo Peng, Ran Xu, Tong Lu, Xiaoyan Chang, Zhiping Shen, Jiaxin Shi, Meifeng Li, Chenghao Wang, Xiang Zhou, Chengyu Xu, Hao Chang, Linyou Zhang

**Affiliations:** ^1^ Department of Thoracic Surgery, The Second Affiliated Hospital of Harbin Medical University, Harbin, China; ^2^ Department of Thoracic Surgery, Ruijin Hospital, Shanghai Jiaotong University School of Medicine, Shanghai, China; ^3^ Department of Thoracic Surgery, The First Affiliated Hospital of Harbin Medical University, Harbin, China

**Keywords:** lung adenocarcinoma, PPP4C, immunotherapy, prognosis, single-cell

## Abstract

**Background:**

Elevated PPP4C expression has been associated with poor prognostic implications for patients suffering from lung adenocarcinoma (LUAD). The extent to which PPP4C affects immune cell infiltration in LUAD, as well as the importance of associated genes in clinical scenarios, still requires thorough investigation.

**Methods:**

In our investigation, we leveraged both single-cell and comprehensive RNA sequencing data, sourced from LUAD patients, in our analysis. This study also integrated datasets of immune-related genes from InnateDB into the framework. Our expansive evaluation employed various analytical techniques; these included pinpointing differentially expressed genes, constructing WGCNA, implementing Cox proportional hazards models. We utilized these methods to investigate the gene expression profiles of PPP4C within the context of LUAD and to clarify its potential prognostic value for patients. Subsequent steps involved validating the observed enhancement of PPP4C expression in LUAD samples through a series of experimental approaches. The array comprised immunohistochemistry staining, Western blotting, quantitative PCR, and a collection of cell-based assays aimed at evaluating the influence of PPP4C on the proliferative and migratory activities of LUAD cells.

**Results:**

In lung cancer, elevated expression levels of PPP4C were observed, correlating with poorer patient prognoses. Validation of increased PPP4C levels in LUAD specimens was achieved using immunohistochemical techniques. Experimental investigations have substantiated the role of PPP4C in facilitating cellular proliferation and migration in LUAD contexts. Furthermore, an association was identified between the expression of PPP4C and the infiltration of immune cells in these tumors. A prognostic framework, incorporating PPP4C and immune-related genes, was developed and recognized as an autonomous predictor of survival in individuals afflicted with LUAD. This prognostic tool has demonstrated considerable efficacy in forecasting patient survival and their response to immunotherapeutic interventions.

**Conclusion:**

The involvement of PPP4C in LUAD is deeply intertwined with the tumor’s immune microenvironment. PPP4C’s over-expression is associated with negative clinical outcomes, promoting both tumor proliferation and spread. A prognostic framework based on PPP4C levels may effectively predict patient prognoses in LUAD, as well as the efficacy of immunotherapy strategy. This research sheds light on the mechanisms of immune interaction in LUAD and proposes a new strategy for treatment.

## 1 Introduction

Lung adenocarcinoma (LUAD), a prevalent form of lung cancer, remains a leading cause of cancer-associated mortality globally (1), displaying a five-year survival rate of merely 15% (2–4). Among the primary therapeutic targets for LUAD are PD-1 and PD-L1 (5–8). The field has seen considerable interest in immune checkpoint blockade (ICB), noted for substantially enhancing survival rates in cancer patients. Despite these advances, the effectiveness of immunotherapy is not universal among LUAD patients (9). Factors such as the PD-1 expression, MSI, TMB, and tumor microenvironment play crucial roles in determining the response to immune treatments (10–13). Nevertheless, the prognostic tools currently available lack precision, underscoring the imperative need for research into vital genes and biomarkers that influence both the prognosis of LUAD patients and the response to immunological therapies (14–17).

Nowadays, high-throughput sequencing tools such as second-generation sequencing (18, 19), single-cell transcriptomics sequencing (20), spatial transcriptomics sequencing, proteomics sequencing, and metabolomics sequencing are rapidly evolving (21). These technological advances have brought new perspectives to the field of genetic research. Utilizing patterns of gene expression, researchers aim to uncover novel biomarkers within various cancers (22, 23). By rigorously analyzing high-throughput RNA sequencing data, it is possible to support initiatives in personalized treatment and precision medicine. This includes identifying emerging prognostic markers and therapeutic targets, elucidating principal genes that affect the immune infiltration status of patients, and delineating the molecular pathways that promote the progression of LUAD (24, 25). These objectives can be fulfilled through the application of differential expression analysis and functional enrichment studies (26).

Protein phosphatases from the PPP family play crucial roles in a variety of physiological and pathological contexts, notably in oncological disorders (27). In particular, the enzyme PPP4C has been linked to the advancement of numerous malignancies, such as breast and pancreatic cancers, and glioblastomas (28). The connection between the expression levels of PPP4C, immune cell infiltration, and the efficacy of chemoimmunotherapy treatments has been established (29–32). However, the specific role of PPP4C within the framework of LUAD has yet to be clarified.

To address the challenge of identifying potential therapeutic targets and prognostic biomarkers for lung adenocarcinoma, our study focused on analyzing the differential immunogenomic profiles in both tumor and non-tumor tissues from LUAD patients. Employing methodologies such as gene set enrichment analysis, survival analysis, WGCNA, single-cell transcriptomics analysis and assessment of differentially expressed immune-related genes, we pinpointed PPP4C as a pivotal subject for further investigation (33). Our research further explored how PPP4C expression within the tumor immune microenvironment influences LUAD prognosis. By constructing a risk assessment model incorporating PPP4C alongside other immunologically relevant genes, we successfully predicted outcomes and immune response efficacy in LUAD therapy. Furthermore, our investigation extended to analyzing clinical and immune profiles across various risk categories, culminating in the development of diagnostic charts that offer novel insights into managing LUAD and evaluating immunotherapy responses in treated patients.

## 2 Materials and methods

### 2.1 Participant recruitment and data collection

Between June and August 2023, individuals undergoing surgical procedures at Harbin Medical University’s Second Affiliated Hospital contributed samples from three distinct pairs of lung adenocarcinoma and adjacent non-malignant tissues. These specimens encompassed paired samples of lung adenocarcinoma tumors and the corresponding adjacent healthy tissues. Authorization for this research was granted by the Ethics Committee of the Second Affiliated Hospital at Harbin Medical University under the protocol number KY2023–042.

Clinical records and RNA sequencing data from 594 LUAD patients were retrieved from the TCGA repository (https://portal.gdc.cancer.gov/). Furthermore, RNA sequencing and survival statistics for an additional 246 LUAD patients were accessed through GEO dataset GSE31210 (34). The dataset GSE123902, which includes RNA sequencing data at the single-cell level, comprised thirteen tumor and four normal tissue samples. An immunotherapy validation cohort was established using data from the R program IMvigor210. Additionally, gene sets pertinent to immune responses were obtained from both InnateDB and ImmPort databases.

### 2.2 Identification of immune-related genes

To ascertain immune-associated genes, differential expression analysis was conducted on LUAD cohort data from the TCGA database, employing the limma software (|log2FC| > 0.585) (35). Subsequently, lists of immune genes sourced from the InnateDB and ImmPort databases were amalgamated to facilitate functional enrichment analysis. Genes within the yellow and brown modules were selected based on a significance threshold of *P* < 0.05. The identification of pivotal genes in immune regulation was achieved through the application of the WGCNA algorithm (33, 36). Functional enrichment analysis was subsequently conducted using the clusterProfiler software (37).

### 2.3 Single-cell RNA sequencing

In this investigation, data from single-cell RNA sequencing was analyzed using the Seurat package within R software (version 4.4.0) (38). Initially, rigorous quality control protocols were employed to eliminate substandard cells, which required the establishment of specific criteria such as nFeature between 300 and 7,500, nCount between 300 and 100,000, mitochondrial gene expression ratio of less than 20%, ribosomal gene expression ratio of greater than 3 and erythrocyte gene expression ratio of less than 0.1. Subsequently, the SCTransform method was applied for data normalization, followed by dimensionality reduction via the RunTSNE function, facilitating easier clustering and visualization. Knowledge from prior studies and the CellMarker database (http://xteam.xbio.top/CellMarker) aided in cell type annotation (39). Furthermore, normal epithelial cells in the sample were used as a control group, and the inferCNV (https://github.com/broadinstitute/inferCNV/) algorithm was utilized to differentiate between benign and malignant epithelial cells based on differences in chromosome copy number variants and to explore the differences in the expression of PPP4C in the two types of cells, Then, tumor cells were categorized according to the median PPP4C expression level, and differential pathway enrichment was explored using GSVA analysis, and CellPhoneDB (version 2.0) elucidated cell-cell interactions, with higher ligand-receptor interaction scores suggesting more robust intercellular communication.

### 2.4 Immune infiltration

LUAD patients were stratified into groups with high and low levels of PPP4C gene expression based on median values. Subsequent analysis of immune cell infiltration differences between these groups employed the CIBERSORT algorithm (40). Comparative immunological assessments utilized the ESTIMATE method (41, 42), alongside Spearman’s rank correlation analysis. To delineate key genetic markers for constructing a prognostic model, the LASSO and univariate regression methodologies isolated 14 critical genes (43, 44). Furthermore, multivariable Cox regression analysis facilitated the establishment of risk scores, categorizing patients into cohorts with elevated or diminished risk profiles.

### 2.5 The immunological landscapes, clinical manifestations, and immunotherapy

To elucidate the interplay between immunological profiles, clinical characteristics, and responses to immunotherapy, our study employed MCPcounter to quantify immune cell infiltration within distinct risk strata. This approach facilitated an exploration of the correlations between risk assessments and clinical manifestations. Furthermore, the ESTIMATE algorithm was applied to evaluate differences in immunological scores across the cohorts (45). We investigated disparities in the expression of genes associated with immune checkpoints across these risk groups. Subsequent analysis focused on contrasting the tumor mutational burden between individuals classified as high-risk and those deemed low-risk, alongside investigating variations in prognostic outcomes, responses to immune treatments, and staging utilizing the R IMvigor210 toolset. In pursuit of developing a predictive framework, we conducted both univariate and multivariate Cox regression analyses. The decision curve analysis (DCA) was subsequently utilized to determine the predictive precision and clinical utility of the developed model.

### 2.6 Real-time PCR

In this investigation, the methodology of real-time quantitative polymerase chain reaction (RT-qPCR) was utilized. For the synthesis of cDNA, cellular RNA was initially isolated using Trizol (Sigma), followed by reverse transcription using PrimeScriptTM RT Master Mix (TaKaRa). Subsequently, the ChamQ Universal SYBR qPCR Master Mix (Vazyme) facilitated the RT-qPCR analysis. The quantification of mRNA expression levels of the target genes was performed utilizing the 2-ΔΔCT approach, wherein β-actin served as the internal standard (46). Data analysis and the creation of graphical outputs were conducted with the aid of Prism software.

The primer sequences used were as follows: for PPP4C, 5’- GGTCTATGGCTTCTACGATG -3’; and for β-actin, 5’-GAAGAGCTACGAGCTGCCTGA-3’.

### 2.7 Western blot

Proteins were extracted from washed tissues using a RIPA buffer enhanced with PMSF and a cocktail of protease inhibitors. Subsequently, protein concentrations were quantified employing the BCA assay. Proteins were resolved by SDS-PAGE and transferred onto PVDF membranes. The membranes underwent a blocking process using 5% non-fat milk before antibody incubation. Primary antibodies were applied overnight at 4°C, followed by a one-hour incubation with secondary antibodies at ambient temperature (47). In this study, the primary antibodies utilized were anti-β-actin and anti-PPP4C, while Goat anti-Rabbit HRB served as the secondary antibody. Visualization of the protein bands on the Western blots was facilitated using an ECL detection system.

### 2.8 Immunohistochemistry

In the course of this study, the immunohistochemistry protocol was meticulously followed through several distinct methodological phases (48). Initially, tissue sections were subjected to a pre-treatment phase within a temperature-controlled oven. Ensuring cellular exposure to the antigen involved multiple steps including the removal of paraffin and the retrieval of antigens. Overnight incubation with primary antibodies, sourced from Abcam, was then conducted. Visualization of the staining process was facilitated using the Dako EnVisionTM FLEX+ kit. Subsequently, an Aperio digital pathology slide scanner was employed to capture the resultant images, and additional staining was performed using hematoxylin provided by Sigma Aldrich. The semi-quantitative analysis of immunohistochemical staining was performed using the IHC Profiler plugin in Image J software, and the IHC score was calculated based on the percentage of area of different staining intensities. (IHC sore = High positive areas percentage × 3 + Positive areas percentage × 2 + Low positive areas percentage × 1 + Negative areas percentage × 0)

### 2.9 Cultivation of lung cancer cells

Cultures of the A549, H1299, PC9 and H1975 cell lines (sourced from PLST Co., Ltd., China) were propagated using RPMI-1640 medium (manufactured by Gibco, USA), which was enriched with 10% fetal bovine serum. BEAS-2B cell line culture was performed in serum-free BEpiCM complete medium (manufactured by SclenCell, USA). These cultures were sustained at a constant temperature of 37°C within a humidified environment, supplemented by an atmosphere containing 5% CO2.

### 2.10 PPP4C influences on the migration and invasion of LUAD cells

In the investigation of PPP4C’s role in inhibiting LUAD cell migration, cells were evenly distributed across six-well plates for a wound healing assay, achieving the necessary confluency. Subsequently, serum-free DMEM was employed to foster cell cultures post-creation of uniform scratches using 200 μl pipette tips. After 24 hours of cell culture, the scratched areas were systematically photographed to monitor the healing progress, thus helping to analyze the migration dynamics of the cells.

To determine the invasive properties of the tumor cells, experiments utilized a 24-well, 8μm Transwell setup (NEST Biotechnology Co. LTD., Wuxi, China). Single-cell suspensions were introduced into the upper compartment at a concentration of 1×10^5 cells per well, with serum-free media above and 10% FBS-enriched media below. Following a 24-hour incubation, the cells underwent fixation with 4% paraformaldehyde post-staining with 1% crystal violet (49). Invasion assessments were conducted using an inverted microscope to capture images from at least three random fields.

### 2.11 Cell colonies formation

To investigate colony formation, 500 cells were uniformly distributed into each well of a six-well plate. These cells were incubated at 37°C for two weeks until distinct colonies, comprising no fewer than 50 cells each, could be discerned microscopically. Post-incubation, colonies were fixed using 4% paraformaldehyde and subsequently stained with 1% crystal violet. Photographs documenting colony growth across all wells were subsequently captured (50).

### 2.12 Statistical analysis

To conduct the statistical evaluations, we utilized versions 4.2.2 and 9.5.1 of Prism and R, respectively. Continuous variables were described using means and standard deviations of independent samples. Survival differences were assessed employing Kaplan-Meier curves and the Log-rank test, alongside Spearman’s correlation coefficient and the t-test for comparisons between two groups. A significance level was established at *P* < 0.05.

## 3 Results

### 3.1 Analysis of differentially expressed immune genes in LUAD

**Figure 1:**
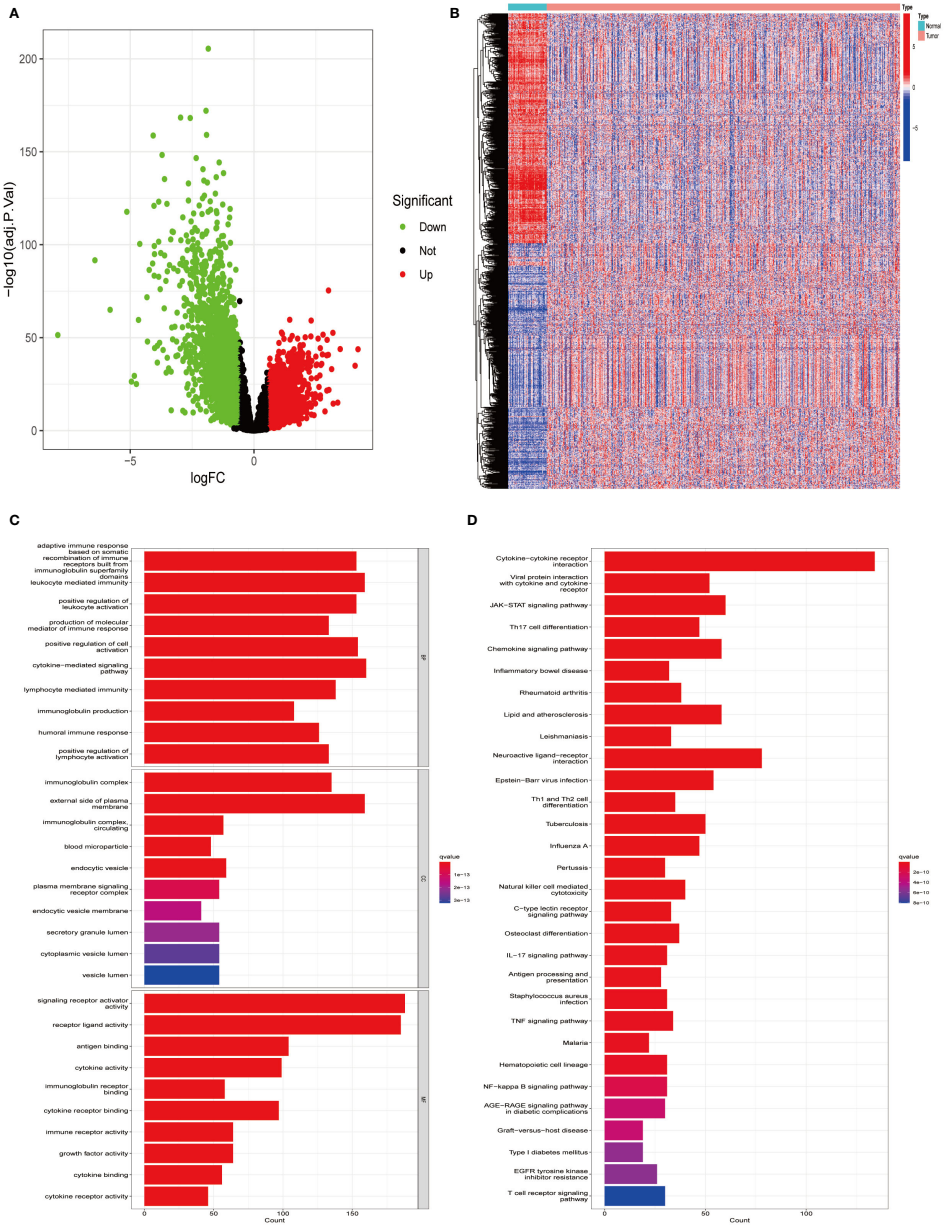
Identifying differentially expressed immune genes in LUAD. **(A)** Comparative analysis was conducted between 535 tumor samples and 59 normal lung tissues from LUAD patients. Up-regulated genes are depicted in red, while down-regulated genes are depicted in green. **(B)** A heatmap visualizes the differential expression pattern of immune-related DEGs between tumor samples (in red) and normal samples (in blue). **(C)** Gene Ontology (GO) enrichment analysis was performed on the identified differential immune genes. **(D)** Kyoto Encyclopedia of Genes and Genomes (KEGG) pathway analysis was conducted on the differential immune genes.

Utilizing the TCGA-LUAD data set for analysis, a survey of gene expression variances unveiled 13,618 genes with fluctuating expression levels. Among these, 4,237 were noted as down-regulated and 9,381 as up-regulated (**Figure 1A**). Subsequent scrutiny against immune-specific databases from InnateDB and ImmPort revealed 1,011 genes linked to immune functionalities showing disparate expression patterns in LUAD, divided into 499 down-regulated and 512 up-regulated genes (**Figure 1B**). Advanced probing into these genes’ roles identified enrichment in 132 KEGG pathways and 2,720 GO terms, with emphasis on the foremost 30 GO terms and KEGG pathways (**Figures 1C, D**).

### 3.2 Characterization of immune-related gene targets in LUAD

**Figure 2:**
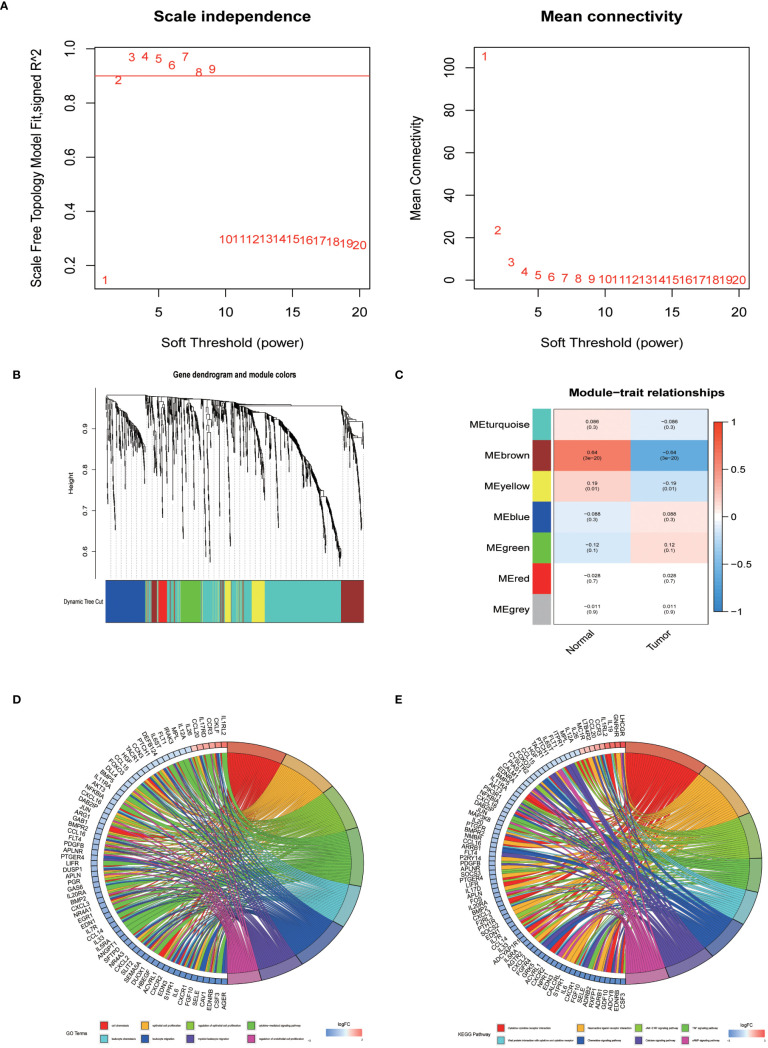
The identification of immune-related hub genes. **(A)** Determination of the soft threshold power through analysis, with the optimal value found to be 2. **(B)** Application of WGCNA on differential immune genes. **(C)** Extraction of seven gene modules through WGCNA. **(D)** GO enrichment analysis of genes within the yellow and brown modules. **(E)** KEGG enrichment analysis of genes within the yellow and brown modules.

In an endeavor to identify pivotal hub genes associated with immunity in lung adenocarcinoma (LUAD), researchers conducted a comprehensive analysis using Weighted Gene Co-expression Network Analysis (WGCNA) on 1011 genes known for their variable expression in immune responses. This analysis constructed a scale-free network, which facilitated the identification of an optimal soft-thresholding power set at 2, as depicted in **Figure 2A**. This specific thresholding power, when applied alongside hierarchical clustering employing average linkage techniques, classified these genes into seven distinct clusters (**Figures 2B, C**). Subsequent analysis calculated Pearson correlation coefficients to link these gene clusters with particular traits of LUAD tumor samples, adopting a significance level of *P* < 0.05. Notably, genes within the brown and yellow clusters were selected for further detailed scrutiny. The intent of this deeper investigation was to elucidate the relationship between immune genes and patient prognosis in LUAD. Prior to a focused analysis of the selected gene clusters, a preliminary survey was conducted to assess their functional involvement and pathway engagement. This led to the revelation of considerable enrichment in the top 8 Kyoto Encyclopedia of Genes and Genomes (KEGG) pathways, shedding light on their biological functions and interconnected roles in LUAD progression (**Figures 2D, E**).

### 3.3 Probing PPP4C expression patterns in lung cancer

**Figure 3:**
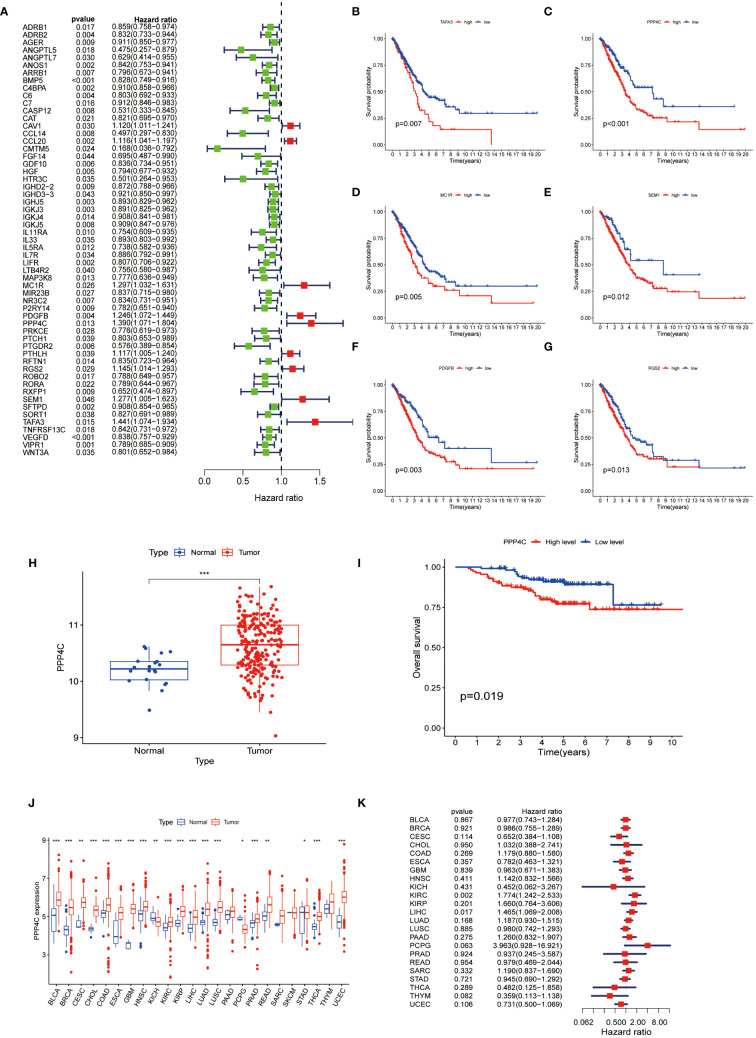
Analysis of PPP4C **(A)** Univariate Cox regression analysis was conducted on 58 immune-related hub genes, where green dots represent protective factors and red dots represent risk factors. **(B-G)** Kaplan-Meier survival analysis was performed on the top six immune-related hub genes with HR>1. **(H)** Differential expression analysis of PPP4C in the GEO cohort. **(I)** Kaplan-Meier survival analysis of PPP4C in the GEO cohort. **(J, K)** Pan-cancer analysis of the PPP4C gene. (*P < 0.05, **P < 0.01, ***P < 0.001).

To ascertain immune-associated genes of independent prognostic value, a meticulous univariate Cox regression coupled with Kaplan-Meier survival analysis was conducted for genes within the brown and yellow modules (**Figures 3A-G**). This analysis brought to light 58 immune genes of prognostic significance. Moreover, earlier investigations have illuminated distinctive expression profiles of the PPP gene family in breast cancer, underscoring its link to both prognosis and the infiltration states of immune cells (21). Notably, PPP4C has been distinguished as a valuable prognostic indicator and as a potential therapeutic target in breast cancer. However, the expression dynamics, biological functions, and impact on immune cell infiltration by PPP4C in lung adenocarcinoma (LUAD) necessitate further inquiry. Thus, our focus has shifted toward the PPP4C gene. Through the analysis of the GSE31210 dataset from the GEO database, we confirmed PPP4C’s unique expression in LUAD and its prognostic ramifications. The findings reveal a significant upregulation of PPP4C in LUAD, correlating with unfavorable outcomes (**Figures 3H, I**). Subsequently, an exhaustive pan-cancer assessment of PPP4C was undertaken to define its biological ramifications across various cancers (**Figure 3J**). This broad evaluation disclosed a uniform pattern of elevated PPP4C expression in numerous cancers, notably associated with adverse results, particularly in patients with liver hepatocellular carcinoma (LIHC) and kidney renal clear cell carcinoma (KIRC) (**Figure 3K**). In conclusion, the pervasive increase in PPP4C expression in LUAD and other cancers highlights its critical role as an indicator of poor prognosis, thereby solidifying PPP4C as a paramount prognostic marker and a prospective therapeutic target.

### 3.4 Delineation of PPP4C expression via single-cell profiling

**Figure 4:**
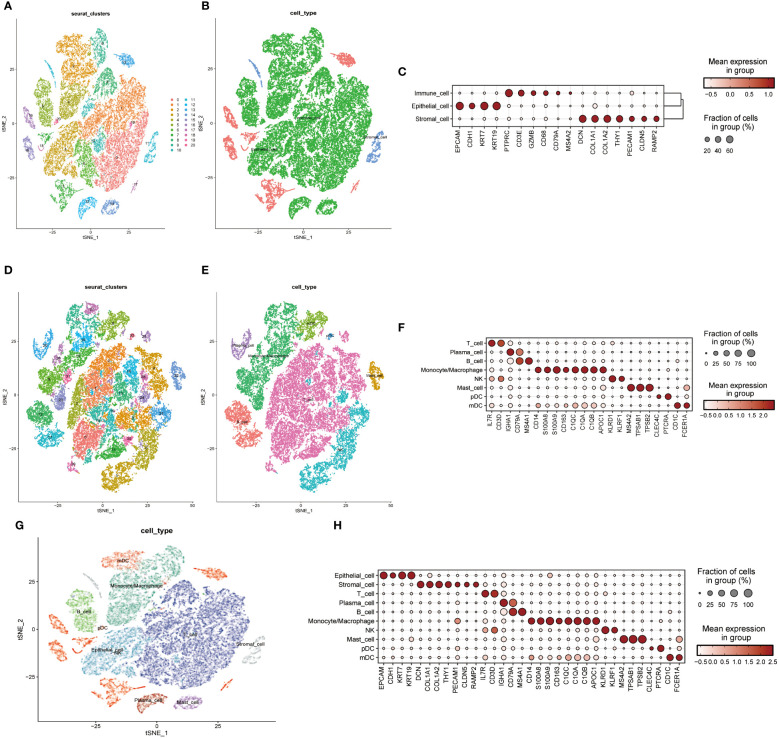
Single-cell mapping. **(A)** t-SNE plots depict all cells in the 20-cell cluster. **(B)** Classification of all cells into three primary types: epithelial, immune, and stromal. **(C)** Demonstration of specific gene expression across different cell types. **(D)** t-SNE plot focusing on immune cell clustering. **(E)** Annotation of the immune cell population into seven major immune cells. **(F)** Presentation of specific gene expression within the seven immune cell types. **(G)** Integration of clustered t-SNE plot for all cells. **(H)** Demonstration of specific gene expression across all cell types.

**Figure 5:**
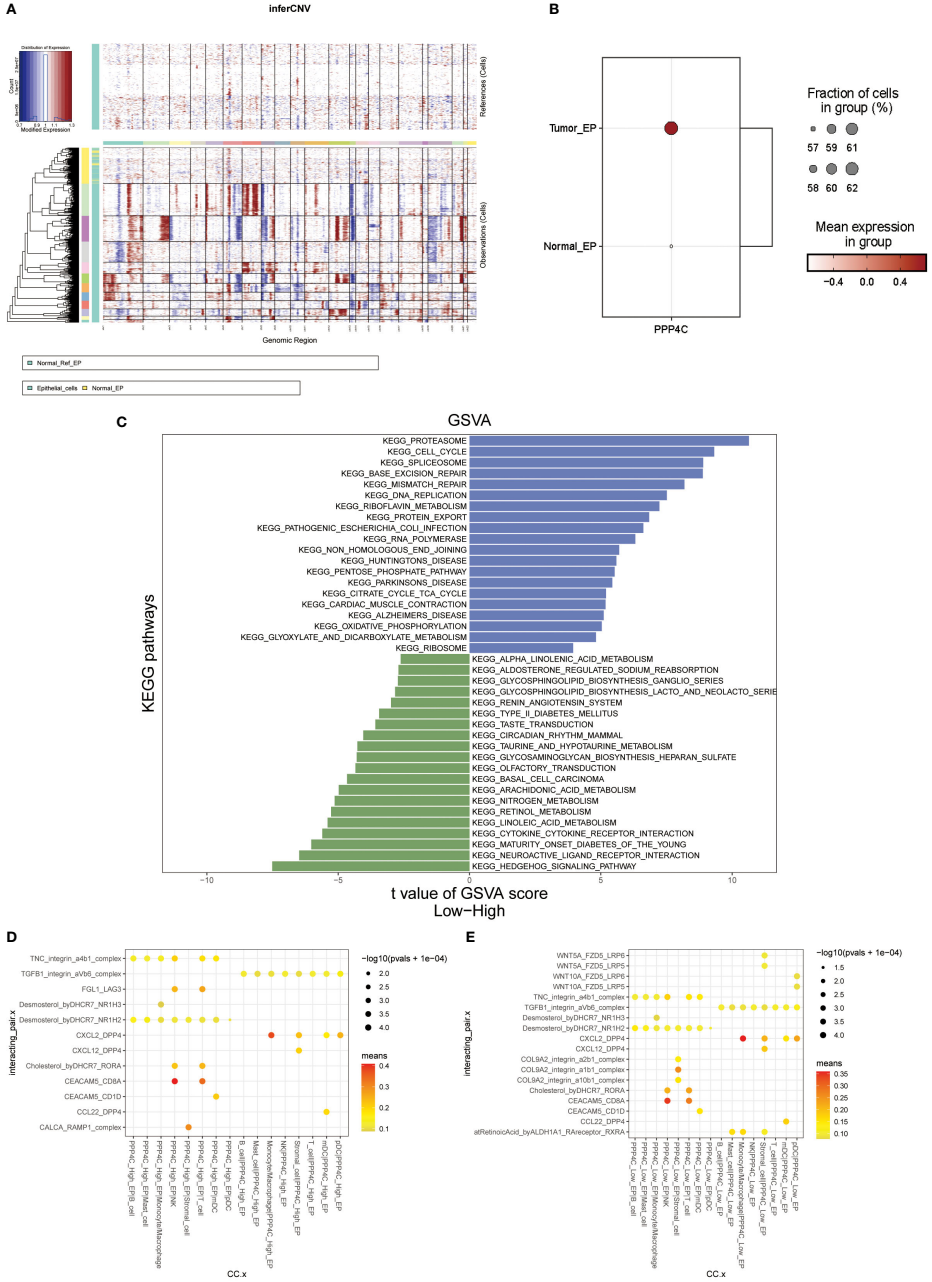
Analysis of functional enrichment. **(A)** Application of the inferCNV algorithm to analyze all epithelial cell copy number variants. **(B)** Comparison of PPP4C differential expression between normal and malignant epithelial cells. **(C)** GSVA pathway enrichment analysis between high and low PPP4C expression groups. **(D)** Investigation of cellular communication between PPP4C-overexpressing malignant epithelial cells and immune cells. **(E)** Examination of cellular communication between PPP4C low-expressing malignant epithelial cells and immune cells.

A single-cell transcriptomic approach to investigating PPP4C expression has uncovered a considerable variation in expression levels and functional roles across cells. By examining the GSE123902 dataset, which encompasses primary, metastatic lung adenocarcinomas, and non-tumorous lung tissues, the study distinguished twenty distinct cellular clusters. These clusters were systematically categorized as epithelial, immune, or stromal cells through integration with the CellMarker database (**Figures 4A-C**). Deeper scrutiny revealed seven principal subtypes within the immune cells (**Figures 4D-F**). The classification strategy hinged on the use of defined cellular markers (**Figures 4G, H**). Subsequent analysis using inferCNV highlighted a conspicuous escalation in PPP4C expression among cancerous epithelial cells (**Figure 5A**), compared to their normal counterparts (**Figure 5B**). Further subdivision of the tumor epithelial cells into high and low PPP4C expression groups, followed by GSVA enrichment analysis, identified significant upregulation of pathways related to the cell cycle, DNA replication, oxidative phosphorylation, and metabolism of glycolysis/TCA cycle in cells with elevated PPP4C expression (**Figure 5C**). Moreover, a comparative analysis of intercellular communication between different factions of cancerous epithelial and immune cells was conducted. This investigation not only casts light on the crucial role of PPP4C in lung adenocarcinoma pathogenesis but also lays the groundwork for further explorations (**Figures 5D, E**).

### 3.5 Exploration of immunological subtypes based on PPP4C gene expression

**Figure 6:**
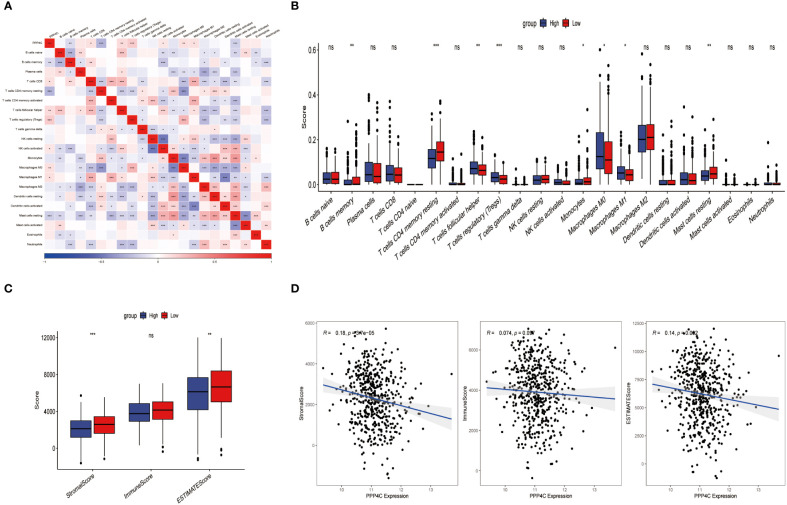
The relationship between PPP4C and immune cell infiltration. **(A)** Correlation analysis between PPP4C gene expression and immune cells. **(B)** Evaluation of differences between PPP4C gene expression and immune cells. **(C)** Assessment of differences between PPP4C gene expression and stromal score, immune score, and ESTIMATE score. **(D)** Correlation of PPP4C gene expression with stromal score, immune score, and ESTIMATE score. (*P < 0.05, **P < 0.01, ***P < 0.001, ns, no significance).

Current research explores the dynamic interaction between PPP4C gene expression and immune cell infiltration, highlighting its importance in modern scientific investigations. This study stratifies patients into categories based on either elevated or reduced PPP4C gene expression levels. Utilizing CIBERSORT software to analyze their sequencing data, the study assesses how PPP4C gene expression impacts the distribution of twenty-two distinct immune cell types (**Figure 6A**). Results reveal that patients with increased PPP4C expression show higher levels of follicular helper T cells, regulatory T cells (Tregs), and both M0 and M1 macrophages. In contrast, individuals with lower expression levels display a higher prevalence of memory B cells, CD4 memory resting T cells, monocytes, and inactive mast cells (**Figure 6B**). Additionally, employing the ESTIMATE algorithm to determine stromal and immune scores within these gene expression subclasses demonstrates a negative correlation between PPP4C gene expression and the immune score (**Figures 6C, D**). Such findings provide crucial insights into the role of the PPP4C gene in immune regulation.

### 3.6 Constructing and validating a predictive model involving PPP4C

**Figure 7:**
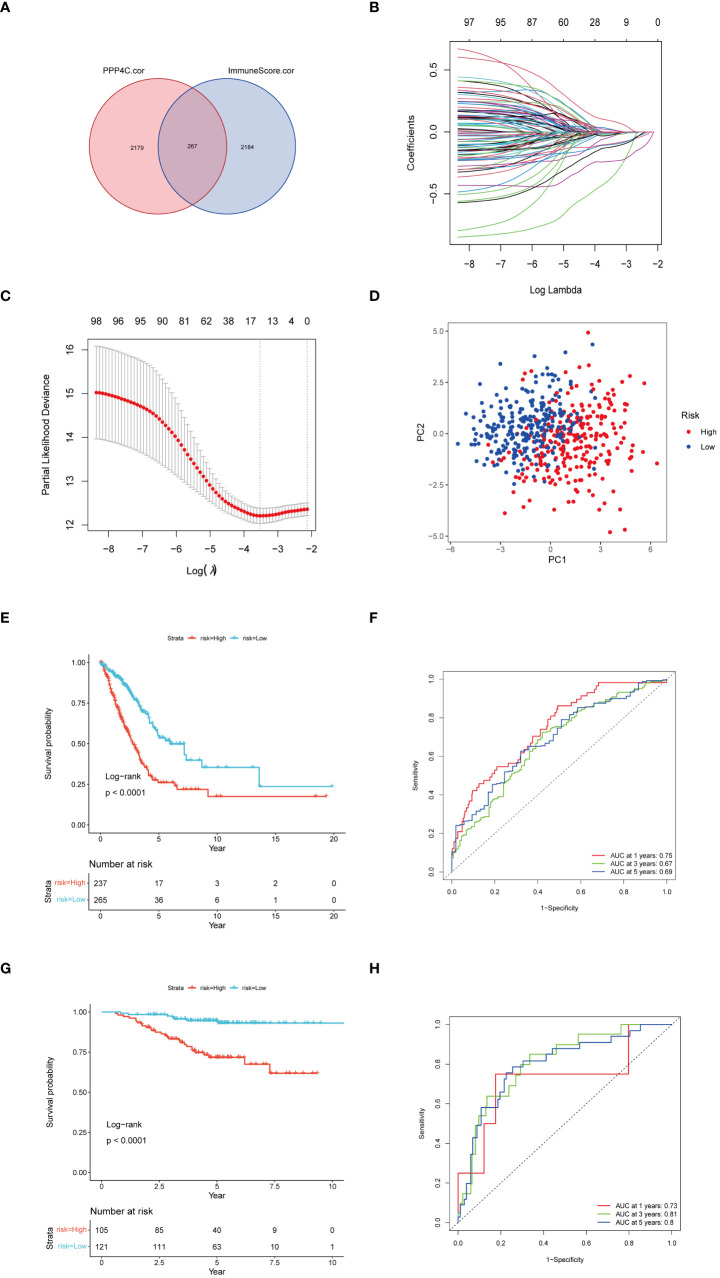
Construction of a risk score model. **(A)** Utilization of a Venn diagram to identify 267 intersecting genes from PPP4C-associated genes and immune score-associated genes. **(B)** LASSO coefficient profiles. **(C)** Determination of the tuning parameter (log λ) based on minimum criteria in the LASSO analysis. **(D)** PCA analysis among different risk groups. **(E)** Kaplan-Meier survival analysis of patients in different risk groups in the TCGA cohort. **(F)** ROC analysis of the TCGA cohort at 1-, 3-, and 5-years. **(G)** Kaplan-Meier survival analysis of patients in different risk groups in the GEO cohort. **(H)** ROC analysis of the GEO cohort at 1-, 3-, and 5-years.

Given the correlation between elevated expression of the PPP4C gene and reduced survival rates in patients with lung adenocarcinoma (LUAD), as well as its ties to immune cell infiltration in the tumor environment, we developed a prognostic model based on PPP4C. This model aims to predict survival and immunotherapy outcomes for LUAD patients. We initially conducted an analysis to identify genes correlated with PPP4C expression and immune parameters, revealing a set of 267 genes (**Figure 7A**). Further enrichment analysis linked these genes to GTPases, pathways of non-small cell lung cancer, PD-L1 expression, and the PD-1 checkpoint, underscoring their potential impact on LUAD prognosis and the efficacy of PD-L1-targeted therapies (**Supplementary Figure S1**). A selected group of 100 genes with significant prognostic value was further narrowed down to 14 key genes through LASSO regression analysis (**Figures 7B, C**). Utilizing these key genes, a risk stratification model was constructed, and risk scores for individual samples were determined via multivariate COX regression analysis. The effectiveness of this model was confirmed through PCA analysis (**Figure 7D**), and the creation of Kaplan-Meier survival and ROC curves demonstrated significantly lower survival in the high-risk group compared to the low-risk group (**Figures 7E, F**). Further validation of the model’s robustness and precision was conducted using a GEO dataset, reinforcing the prognostic model’s accuracy and dependability in predicting survival and responses to immunotherapy in LUAD patients (**Figures 7G, H**).

### 3.7 Assessing the clinical impact of a PPP4C-centric prognostic framework

**Figure 8:**
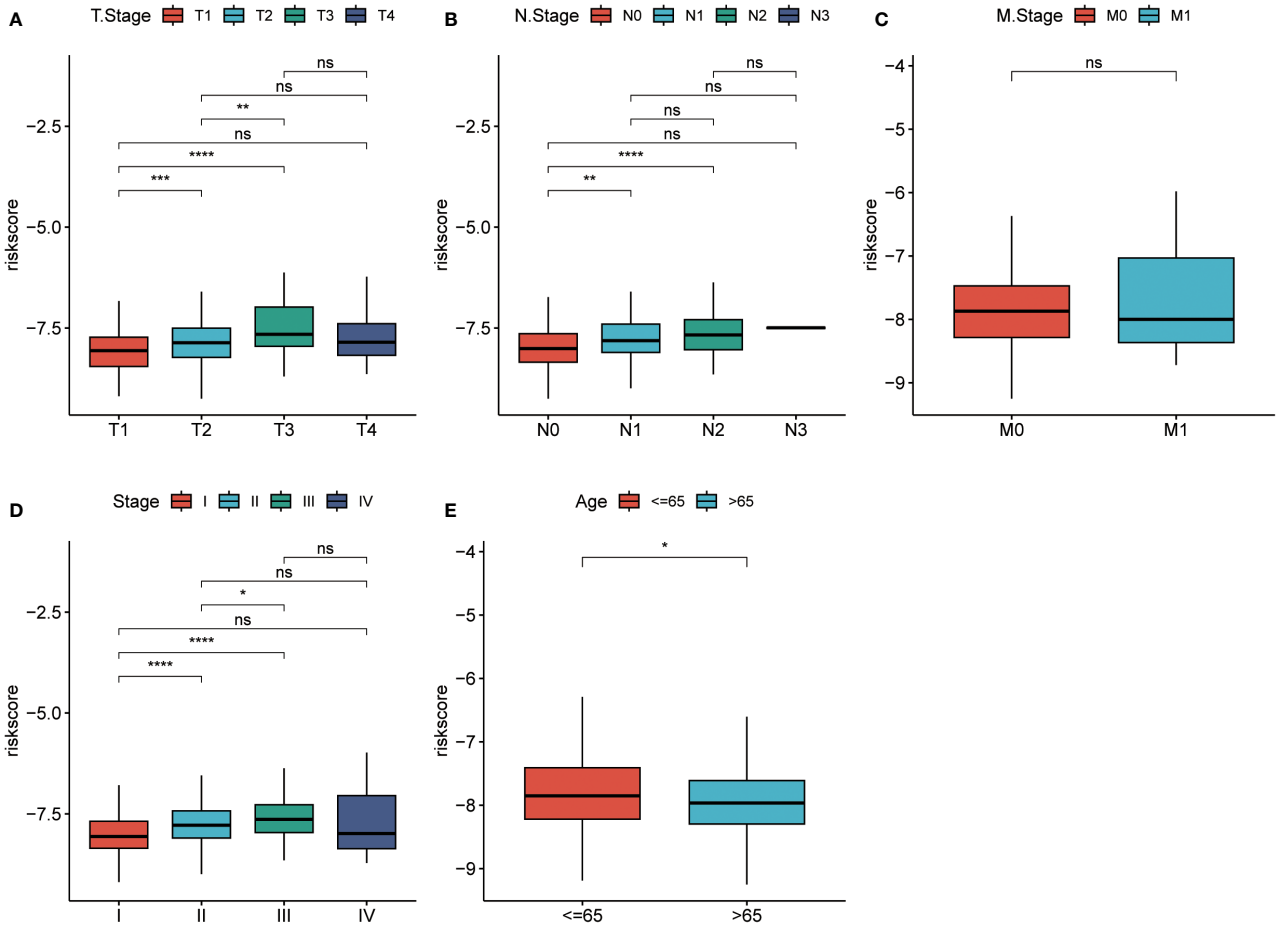
Risk scores in different groups. **(A-C)** T-stage, N-stage, and M-stage. **(D, E)** Pathological stage and age in the TCGA cohort. (*P < 0.05, **P < 0.01, ***P < 0.001, ****P < 0.0001, ns, no significance).

**Figure 9:**
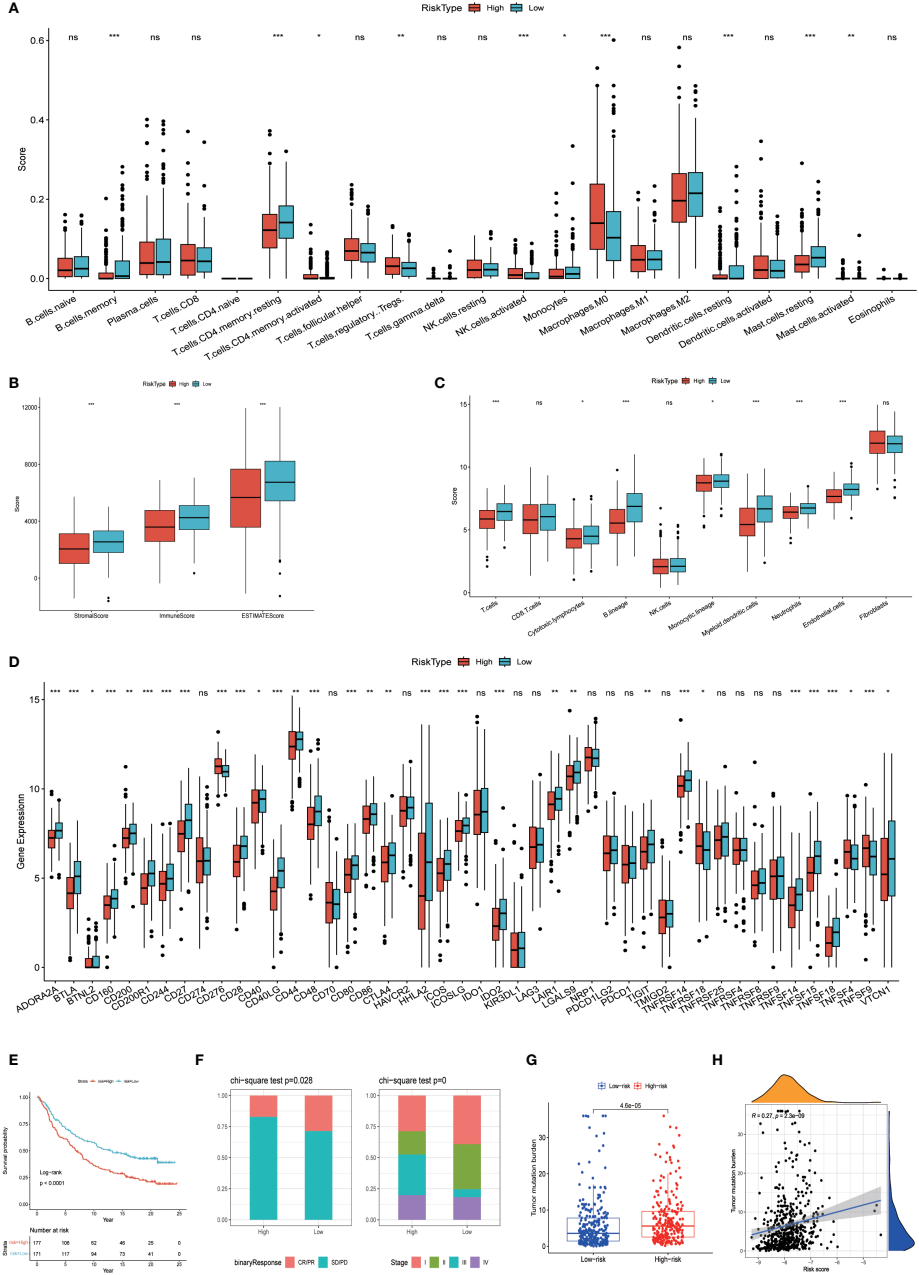
Variations among immune characteristics. **(A)** Illustration of immune cell infiltration differences between patients in different risk subgroups. **(B)** Comparison of immune score differences between patients in different risk subgroups. **(C)** Calculation of differences in immune cell abundance among different risk subgroups of patients based on MCPcounter. **(D)** Evaluation of immune checkpoint-associated gene expression differences among patients in different risk groups. **(E, F)** Kaplan-Meier survival analysis and immunotherapy response in patients from different risk groups of the IMvigor210 cohort. **(G, H)** Analysis of tumor mutation burden differences and correlation in the TCGA cohort of patients. (*P < 0.05, **P < 0.01, ***P < 0.001, ns, no significance).

The prognostic model based on PPP4C demonstrates significant potential for predicting survival rates in patients with lung adenocarcinoma. This approach involves stratifying patients into distinct groups based on clinical features including TNM classification and age. The analysis aims to explore the relationship between these clinical variables and prognostic scores. Results indicate a positive correlation between higher prognostic scores and advanced T, N (**Figures 8A, B**), and pTNM stages (**Figures 8C, D**), particularly in patients under the age of 65 (**Figure 8E**). An extensive analysis was conducted to assess differences in immune cell composition among cohorts displaying varied prognostic scores. Notably, the high-risk group exhibited a reduced presence of T cells CD4 memory activated, Tregs, activated NK cells, and M0 macrophages compared to the low-risk group, which presented an inverse pattern (**Figure 9A**). The variation in immune cell and score metrics among these groups was evaluated using the ESTIMATE algorithm and MCP counting method (**Figures 9B, C**). Furthermore, an analysis comparing immune checkpoint gene expression across different prognostic groups was undertaken (**Figure 9D**). The model’s accuracy in predicting outcomes after immunotherapy was validated (**Figure 9E**), with the high-risk group showing a decreased likelihood of favorable response (**Figure 9F**). The study also included an examination of the disparity in tumor mutation burden between high and low-risk groups (**Figures 9G, H**). These findings underscore the critical clinical importance of utilizing a PPP4C-focused prognostic model for lung adenocarcinoma patients, providing key insights for optimizing therapeutic strategies.

### 3.8 Formulation of a nomogram for precise prognosis prediction in LUAD patients

**Figure 10:**
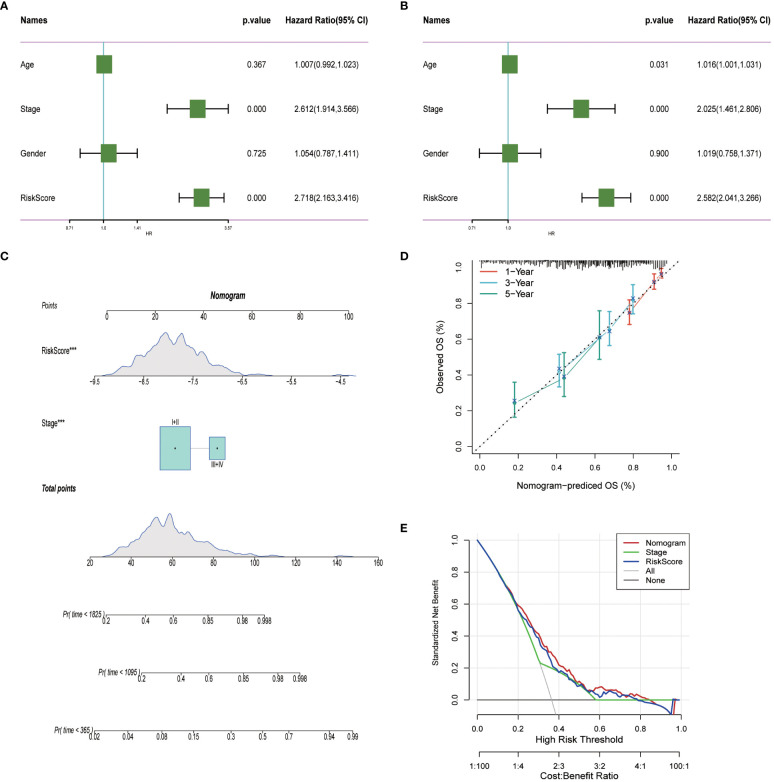
Nomogram in LUAD. **(A, B)** Univariate and multivariate cox regression analysis of risk scores and clinicopathological characteristics. **(C)** Construction of a nomogram combining risk score and clinicopathological staging. **(D)** Calibration curves for 1-, 3- and 5-years for Nomogram. **(E)** Decision curve for nomogram. ***P< 0.001.

To enhance the precision of predictive frameworks in evaluating patient risks, our research integrated clinical-pathological factors with risk scores utilizing both single-factor and multifactorial Cox regression analyses, considering elements like patient demographics. This examination demonstrated that in lung adenocarcinoma (LUAD) scenarios, the derived risk score is an essential independent prognostic factor (P < 0.001), whereas the clinical-pathological stage was of secondary importance (**Figures 10A, B**). Subsequently, a Nomogram was developed, merging both staging data and risk assessments (**Figures 10C**). The prognostic accuracy of this Nomogram was subsequently confirmed through methods of calibration and decision curve analysis (DCA). Calibration diagrams confirmed the Nomogram’s ability to accurately reflect the actual survival rates of patients across timeframes of 1, 3, and 5 years (**Figures 10C**). Moreover, the DCA underscored that the Nomogram markedly surpasses either the risk scores or the pathological staging alone in identifying high-risk patients, thus delivering an enhanced net clinical benefit.

### 3.9 Investigating the impact of PPP4C gene expression on cellular dynamics in LUAD

**Figure 11:**
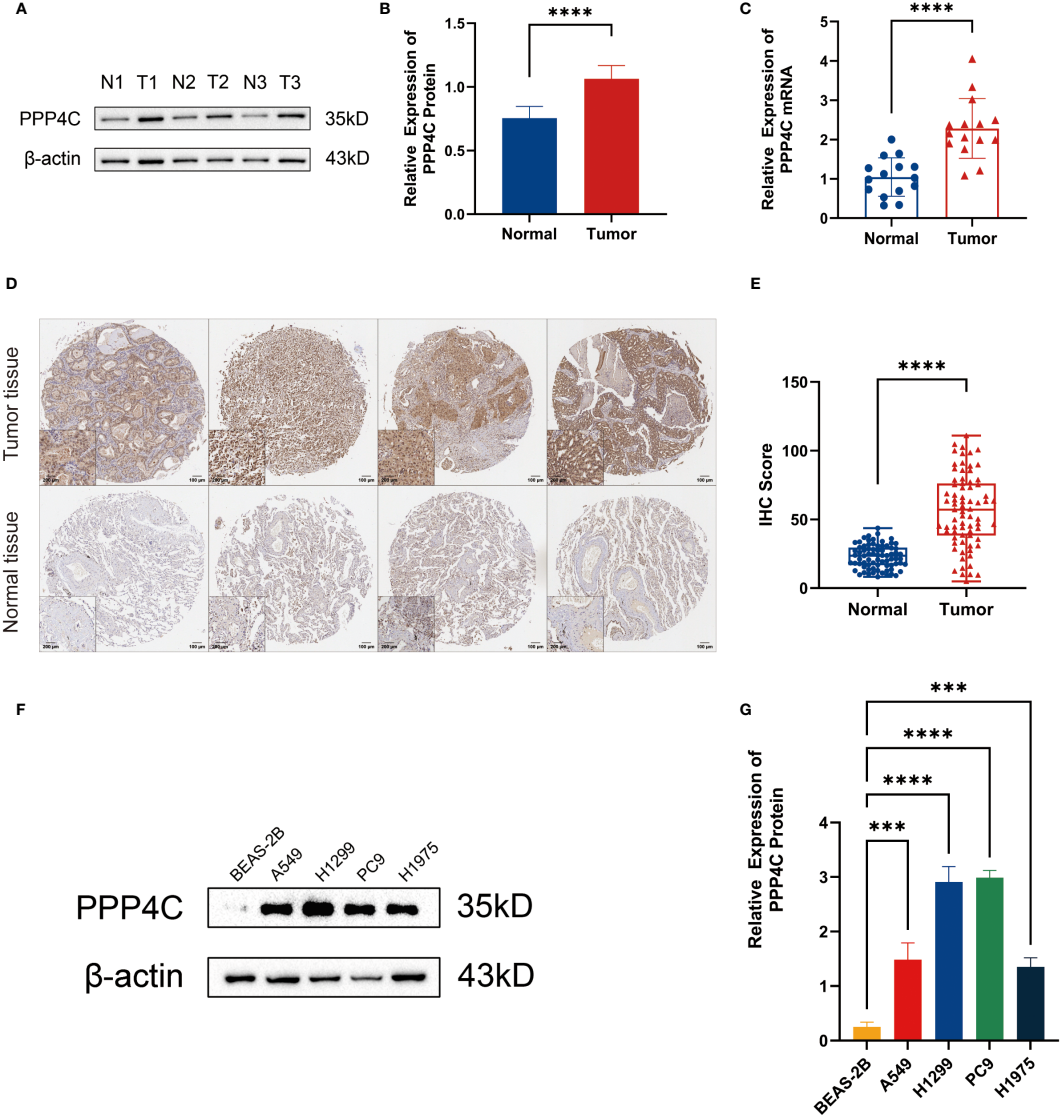
Expression of PPP4C in tumor tissues and normal tissues. **(A, B)** Western blot detection of PPP4C expression in lung adenocarcinoma tissue and normal tissue. **(C)** PCR detection of mRNA expression levels of PPP4C in tumor and normal tissues. **(D, E)** IHC staining and quantitative analysis of lung adenocarcinoma tissues and normal tissues. **(F, G)** Western blotting to detect differences in PPP4C expression in human normal lung epithelial cells BEAS-2B and four types of human lung adenocarcinoma cells A549, H1299, PC9 and H1975. (***P < 0.001, ****P < 0.0001).

**Figure 12:**
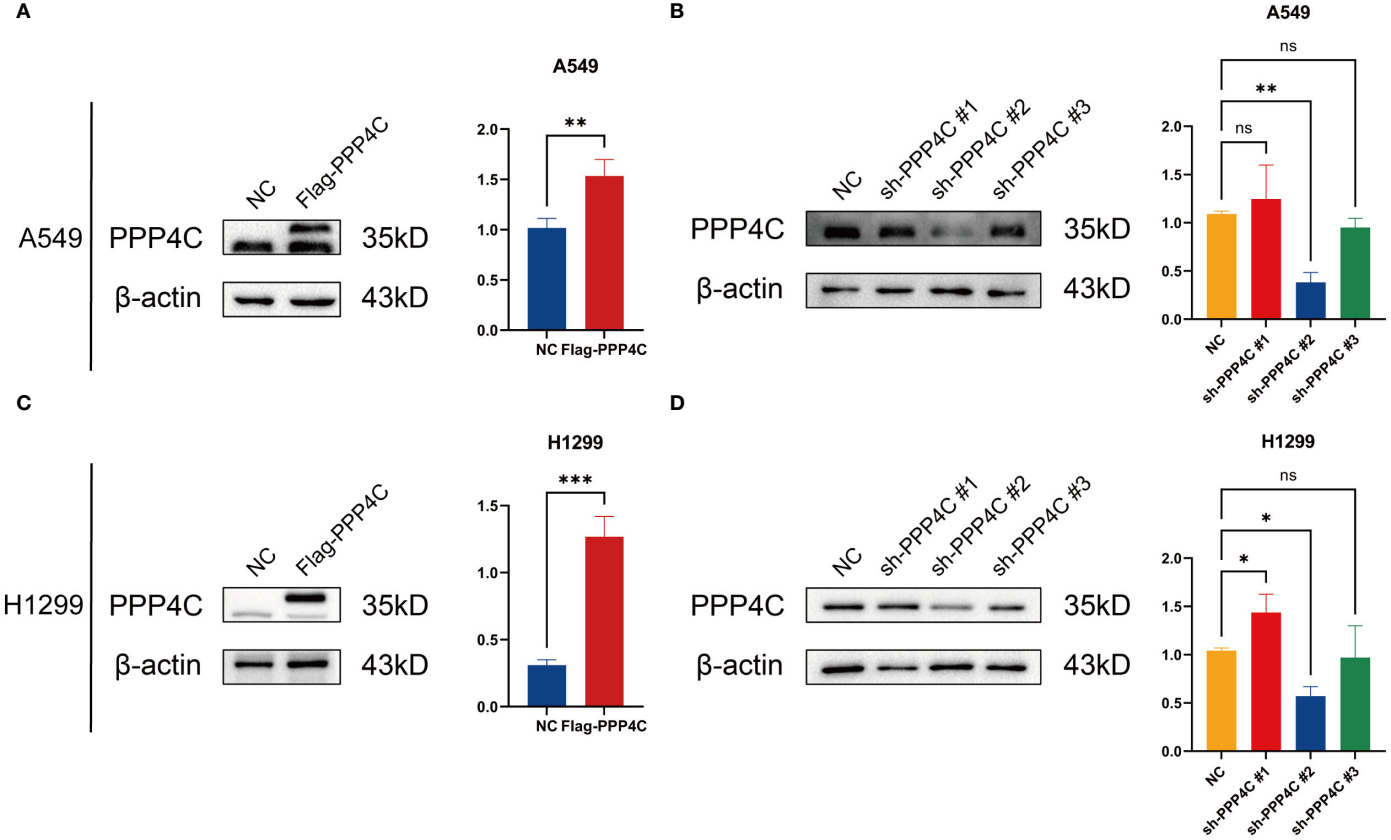
PPP4C overexpression and knockdown in lung cancer cells. **(A, C)** PPP4C overexpression in A549 and H1299 cell lines. **(B, D)** PPP4C knockdown experiment in A549 and H1299 cell lines. (*P < 0.05, **P < 0.01, ***P < 0.001, ns, no significance).

**Figure 13:**
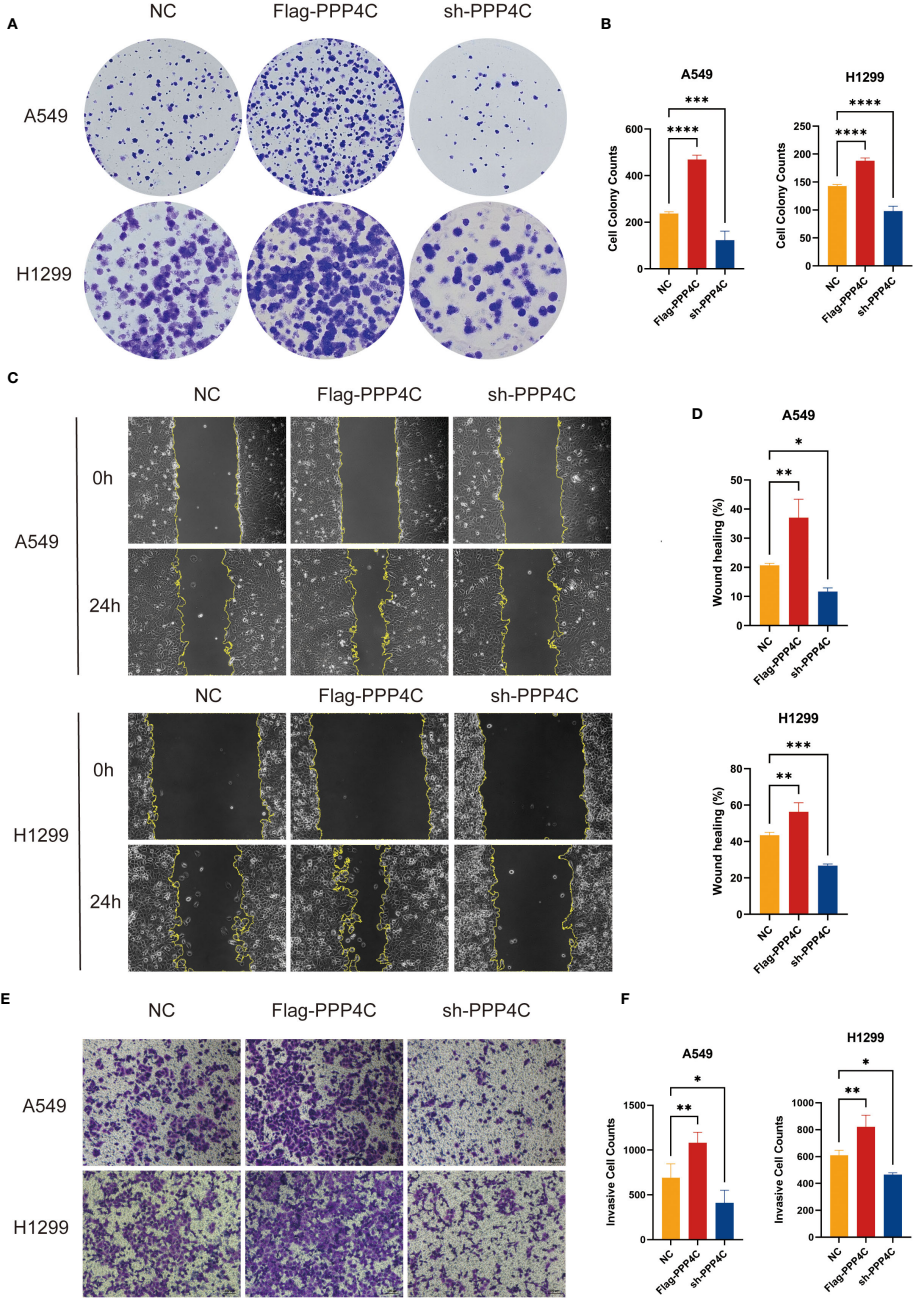
Experimental validation of PPP4C function in lung adenocarcinoma cell line. **(A, B)** Colony formation assay showed that the proliferation ability of A549 and H1299 cells with PPP4C overexpression was significantly enhanced, while the proliferation ability of cells in the PPP4C knockdown group was significantly decreased. **(C, D)** The wound healing assay showed that the cell migration ability was significantly increased in the PPP4C overexpression group compared with the control group, whereas it was significantly reduced in the knockdown group. **(E, F)** Transwell assay showed that the invasive ability of cells in the PPP4C overexpression group was significantly elevated, in contrast to a significant decrease in the invasive ability of cells in the knockdown group. (*P < 0.05, **P < 0.01, ***P < 0.001).

In investigating lung adenocarcinoma, a comprehensive study was conducted to assess the differential expression of PPP4C and its effects on cellular dynamics. The initial stage involved quantifying PPP4C expression in lung adenocarcinoma tissue as well as in adjacent non-cancerous tissue, utilizing methods such as Western blot analysis (**Figures 11A, B**), RT-qPCR (**Figure 11C**), and immunohistochemistry (**Figures 11D, E**). The data revealed a pronounced elevation of PPP4C levels in the tumor tissues compared with the normal tissues, confirmed at both protein and mRNA levels. Meanwhile, we also compared the expression of PPP4C in normal lung epithelial cell lines and lung adenocarcinoma cell lines by Western blot experiments. The results showed that PPP4C was significantly overexpressed in all four lung adenocarcinoma cell lines, which also laid the foundation for our subsequent cell function experiments (**Figures 11F, G**). Subsequent experiments across various lung adenocarcinoma cell lines were designed to clarify the role of PPP4C. By creating cellular models that either overexpressed (**Figures 12A, C**) or suppressed PPP4C expression (**Figures 12B, D**), it was observed that increased PPP4C levels markedly enhanced the proliferation (**Figures 13A, B**), and also the migration and invasion capabilities of A549 and H1299 cells (**Figures 13C, E**). Conversely, a reduction in PPP4C expression led to decreased these cellular processes (**Figures 13D, F**). These results underscore the pivotal influence of PPP4C in lung adenocarcinoma progression and suggest its potential association with patient prognosis.

## 4 Discussion

PPP4C, a member of the phosphatase enzyme family, demonstrates widespread expression across diverse human tissues, suggesting its implication in crucial biological processes. Its conservation throughout evolution underscores its putative involvement in fundamental physiological mechanisms (51). Prior investigations have noted an upregulation of PPP4C expression in numerous cancer types, including colorectal, breast, and pulmonary malignancies (52–55). Within the oncogenic landscape, heightened PPP4C expression correlates with the modulation of pivotal signaling cascades such as mTOR, JNK, and NF-κB, while its depletion may instigate cellular apoptosis (56–58). Notably, PPP4C-mediated augmentation of the ERK pathway fosters lung cancer cell proliferation and impedes apoptotic mechanisms, thereby exacerbating clinical prognosis (59). These insights underscore the potential of PPP4C as a promising therapeutic target across diverse cancer types. Nonetheless, further investigation is imperative to delineate the impact of PPP4C on the immune milieu in lung adenocarcinoma and elucidate its interplay with other oncogenic determinants.

This investigation employed diverse methodologies, encompassing RNA sequencing data analysis, weighted gene co-expression network analysis (WGCNA), and single-cell transcriptome sequencing, to identify pivotal genes within patients afflicted with LUAD and assess their impact on the tumor’s immune milieu. The study revealed a conspicuous escalation in the levels of PPP4C gene among individuals with LUAD, a phenomenon associated with an unfavorable prognosis, a conclusion substantiated by experimental verification. Moreover, an exploration into the nexus between PPP4C gene expression and the tumor’s immune milieu was conducted. The results elucidate a direct correlation between heightened PPP4C expression and diminished immune scores, underscored by a negative correlation between PPP4C expression and immune score. Furthermore, an augmented presence of regulatory T cells (Tregs) was observed in specimens exhibiting elevated PPP4C expression. The dampening of immune response by Tregs emerges as a pivotal tactic employed by tumors to evade immune surveillance, a factor potentially underpinning the adverse prognosis for LUAD patients with heightened PPP4C expression (60). These revelations furnish crucial insights into comprehending the role of PPP4C in LUAD and its ramifications for therapeutic interventions.

Given the significance of PPP4C in prognosis and its impact on the tumor immune microenvironment, we have devised a risk evaluation framework integrating PPP4C with genes associated with immune scoring. This framework serves as a robust prognostic tool, providing insights into patients’ probable response to immunotherapy. Integrating this risk assessment with conventional TNM classifications substantially enhances the predictive capacity of the model. Our investigation encompasses 14 genes pivotal in lung adenocarcinoma (LUAD). Notably, DOCK4 emerges as a suppressor of tumor growth by modulating tumor cell adhesion and invasiveness (61). Moreover, EFHD2 garners attention for its indispensable role in activating immune cells and promoting cancer dissemination (62). METTL7A and MT2A, among other genes, correlate with the prognostic landscape of LUAD patients, implicating their potential impact on disease outcomes (63–65). These revelations underscore a nuanced interplay between these genes and PPP4C’s involvement in LUAD, underscoring the necessity for further exploration to elucidate their functions.

In summary, this study elucidates key genetic markers that impact prognostic outcomes and the immunological microenvironment in patients diagnosed with Lung Adenocarcinoma (LUAD) by integrating single-cell analysis and multi-omics strategies. And the regulatory effect of PPP4C on lung adenocarcinoma cells was verified by differential expression assay and cell function assay. These findings pave the way for identifying new therapeutic targets and prognostic indicators for managing LUAD. Despite the reliance on publicly available datasets, these findings provide valuable insights for further investigations into the oncological significance of PPP4C. Future studies should consider integrating advanced techniques such as machine learning algorithms, spatial transcriptome analysis, and further experimental validation to unravel the biological mechanisms of PPP4C in cancer. These efforts aim to strengthen the basis of precision oncology.

## 5 Conclusion

In patients diagnosed with LUAD, a robust correlation has been established between the levels of PPP4C and the intricacies of the tumor microenvironment’s immune landscape. Elevated PPP4C concentrations serve as harbingers of unfavorable prognostic outcomes, thereby fostering the proliferation and metastasis of lung carcinoma cells. The development and implementation of a risk assessment paradigm centered on PPP4C afford precise prognostic capabilities for individuals afflicted with LUAD, concurrently facilitating the meticulous evaluation of the efficacy of immunotherapeutic interventions. This scholarly inquiry has not only elucidated the intricate pathways governing immune responses in LUAD but also delineated a strategic blueprint for the efficacious management of cancer.

## Data availability statement

The datasets presented in this study can be found in online repositories. The names of the repository/repositories and accession number(s) can be found in the article/**Supplementary Material**.

## Ethics statement

The studies involving humans were approved by The Ethics Committee of the Second Affiliated Hospital of Harbin Medical University. The studies were conducted in accordance with the local legislation and institutional requirements. The participants provided their written informed consent to participate in this study. Written informed consent was obtained from the individual(s) for the publication of any potentially identifiable images or data included in this article.

## Author contributions

KW: Data curation, Formal analysis, Investigation, Methodology, Software, Validation, Visualization, Writing – original draft. BP: Data curation, Formal analysis, Software, Visualization, Writing – review & editing. RX: Data curation, Formal analysis, Software, Visualization, Writing – review & editing. TL: Data curation, Formal analysis, Software, Visualization, Writing – review & editing. XC: Data curation, Software, Visualization, Writing – review & editing. ZS: Data curation, Software, Visualization, Writing – review & editing. JS: Data curation, Software, Visualization, Writing – review & editing. ML: Investigation, Resources, Writing – review & editing. CW: Investigation, Resources, Writing – review & editing. XZ: Investigation, Resources, Writing – review & editing. CX: Investigation, Resources, Writing – review & editing. HC: Conceptualization, Investigation, Supervision, Validation, Writing – review & editing. LZ: Conceptualization, Funding acquisition, Investigation, Methodology, Project administration, Resources, Supervision, Validation, Writing – review & editing.

## Supplementary material

Supplementary Figure 1 Enrichment analysis of PPP4C and immune score intersection genes. **(A-C)** GO enrichment analysis of intersecting genes (P <0.05). **(D)** KEGG enrichment analysis of intersecting genes (P <0.05).
