# Supplementary figures and images for: Comprehensive analysis of PPP4C’s impact on prognosis, immune microenvironment, and immunotherapy response in lung adenocarcinoma using single-cell sequencing and multi-omics
Source: Front Immunol. 2024 Jul 4;15:1416632. doi: 10.3389/fimmu.2024.1416632 (PMC11254641; doi:10.3389/fimmu.2024.1416632)

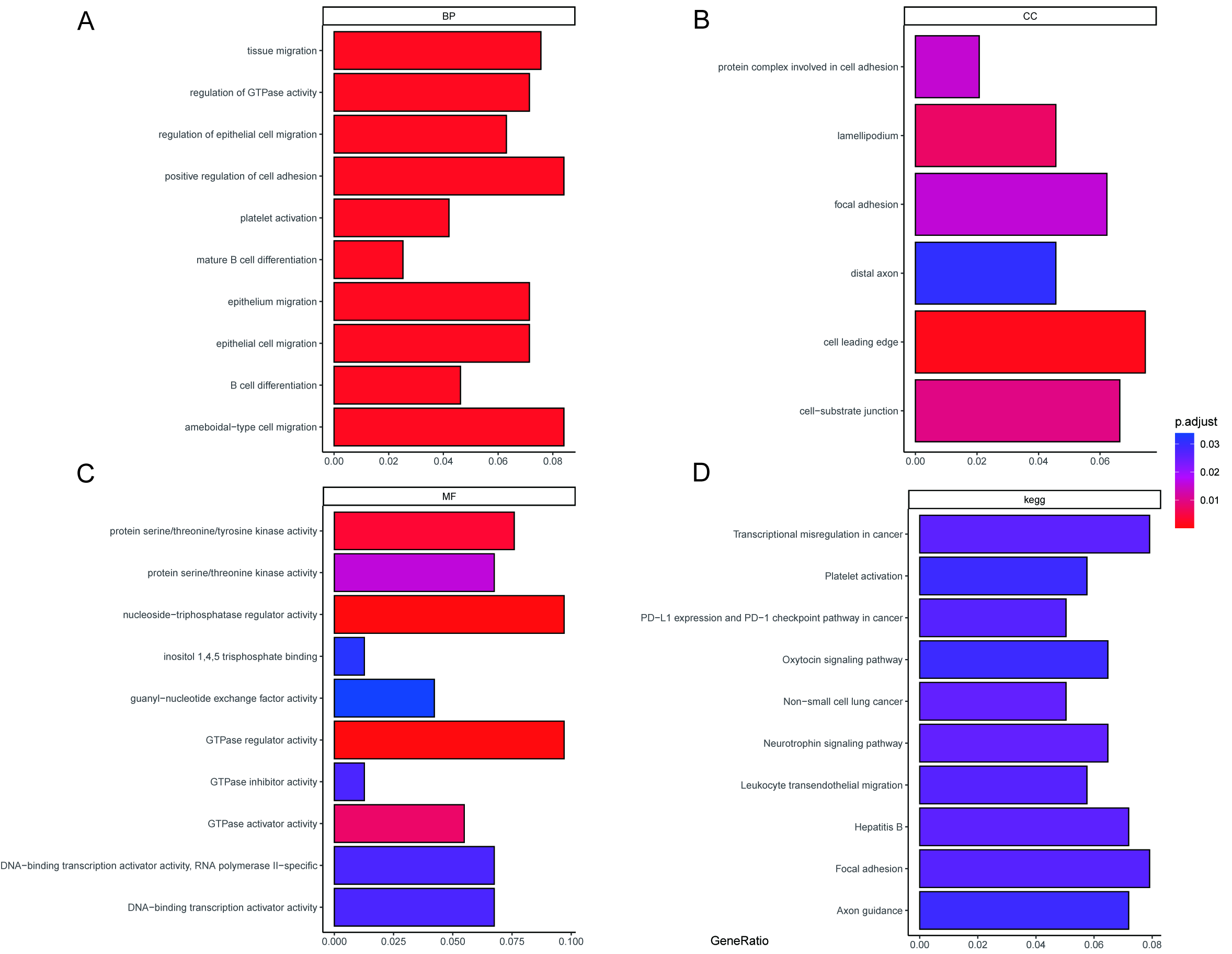

Supplement: Supplementary Figure 1 — Enrichment analysis of PPP4C and immune score intersection genes. (A-C) GO enrichment analysis of intersecting genes (P <0.05). (D) KEGG enrichment analysis of intersecting genes (P <0.05). [file Image_1.tif]
